# Genome-Wide Detection of Predicted Non-coding RNAs Related to the Adhesion Process in *Vibrio alginolyticus* Using High-Throughput Sequencing

**DOI:** 10.3389/fmicb.2016.00619

**Published:** 2016-04-28

**Authors:** Lixing Huang, Jiao Hu, Yongquan Su, Yingxue Qin, Wendi Kong, Lingmin Zhao, Ying Ma, Xiaojin Xu, Mao Lin, Jiang Zheng, Qingpi Yan

**Affiliations:** ^1^Key Laboratory of Healthy Mariculture for the East China Sea, Ministry of Agriculture, Fisheries College, Jimei UniversityXiamen, China; ^2^College of Ocean and Earth Sciences, Xiamen UniversityXiamen, China; ^3^State Key Laboratory of Large Yellow Croaker BreedingNingde, China

**Keywords:** transcriptome, ncRNA, *Vibrio alginolyticus*, adhesion

## Abstract

The ability of bacteria to adhere to fish mucus can be affected by environmental conditions and is considered to be a key virulence factor of *Vibrio alginolyticus*. However, the molecular mechanism underlying this ability remains unclear. Our previous study showed that stress conditions such as exposure to Cu, Pb, Hg, and low pH are capable of reducing the adhesion ability of *V. alginolyticus.* Non-coding RNAs (ncRNAs) play a crucial role in the intricate regulation of bacterial gene expression, thereby affecting bacterial pathogenicity. Thus, we hypothesized that ncRNAs play a key role in the *V. alginolyticus* adhesion process. To validate this, we combined high-throughput sequencing with computational techniques to detect ncRNA dynamics in samples after stress treatments. The expression of randomly selected novel ncRNAs was confirmed by QPCR. Among the significantly altered ncRNAs, 30 were up-regulated and 2 down-regulated by all stress treatments. The QPCR results reinforced the reliability of the sequencing data. Target prediction and KEGG pathway analysis indicated that these ncRNAs are closely related to pathways associated with *in vitro* adhesion, and our results indicated that chemical stress-induced reductions in the adhesion ability of *V. alginolyticus* might be due to the perturbation of ncRNA expression. Our findings provide important information for further functional characterization of ncRNAs during the adhesion process of *V. alginolyticus*.

## Introduction

*Vibrio alginolyticus* is a ubiquitous organism in seawater, and this bacterium has been isolated from different marine organisms as part of the saprophytic microbiota (Carli et al., 1993). *V. alginolyticus* is one of the most important opportunistic pathogens of marine fish and is commonly associated with epidemic vibriosis, which causes mass mortality to cultured *Pseudosciaena crocea* (Wang et al., 2001; Yan et al., 2001). The ability of bacteria to adhere to fish mucus is considered to be a key virulence factor (Chen et al., 2008; Winkelstroeter et al., 2011; Benhamed et al., 2014; Kumaran et al., 2014). Although this ability can be affected by environmental conditions, the underlying molecular mechanism remains unclear.

Bacterial ncRNAs are an emerging class of small regulatory RNAs (<500 nt in length) that play a variety of important roles in many biological processes by binding to mRNA or protein targets (Cao et al., 2010). In addition to RNAs with housekeeping functions, in-depth analysis of several ncRNAs has led to the discovery of various novel regulatory functions. These functions modulate a wide range of responses to stresses and other environmental stimuli (Waters and Storz, 2009), including RNA processing, RNA degradation and translation control.

Interestingly, ncRNAs may interact with other regulatory mechanisms, such as the two-component signal transduction systems, chemotaxis systems, quorum sensing systems, and second messengers, directly and/or indirectly, to form regulatory networks to regulate the switch of swimming and surface attachment. And more interestingly, the same ncRNAs may have opposite roles in different but related bacteria. For example, although the Qrr sRNAs seemingly participate in similar regulatory pathways in *V. cholerae* and *V. harveyi*, the collective actions (redundant versus additive) of the involved Qrr sRNAs and their ultimate effects (stimulation versus inhibition) on biofilm formation are completely different (Dang and Lovell, 2016).

ncRNAs have been shown to regulate a wide variety of biological processes, including secretion, quorum sensing, stress responses, biofilm formation, and virulence (Mann et al., 2012; Jorgensen et al., 2013). In addition, certain ncRNAs have been demonstrated to play a key role in the process of bacterial adhesion. For example, Mann et al. (2012) observed that DF20 and DF32/tmRNA decreased the adhesion of *Streptococcus pneumoniae* to nasopharyngeal and endothelial cells, respectively (Mann et al., 2012). The OmpA protein is utilized by *Escherichia coli* for adhesion to HeLa epithelial cells and Caco-2 colonic epithelial cells, and the VrrA RNA has been shown to affect *V. cholerae* virulence by regulating the expression of OmpA and TcpA (Song et al., 2008). TcpA is the major subunit of an intestinal colonization factor of *V. cholerae* known as the toxin co-regulated pilus (TCP). Additionally, VrrA is the first ncRNA known to control outer membrane vesicle (OMV) formation, which has been suggested to promote the adherence, the transfer of bacterial DNA and the delivery of virulence factors to bacterial or eukaryotic cells (Kuehn and Kesty, 2005; Mashburn-Warren and Whiteley, 2006). However, little is known about *V. alginolyticus* ncRNAs and their potential regulatory functions in adhesion.

Here, we present the first deep sequencing investigation of ncRNAs in *V. alginolyticus* cultured under normal and stress conditions (Cu, Pb, Hg, and low pH). The objectives of this study were to gain a broad-spectrum view of the expression of potential ncRNAs associated with bacterial adhesion and to provide insight to further our understanding of the mechanism(s) underlying the regulation of *V. alginolyticus* adhesion capacity.

## Materials and Methods

### Bacterial Samples and Culture Conditions

Pathogenic *V. alginolyticus* (ND-01) was previously isolated by our lab from naturally infected large yellow croakers and confirmed to be a pathogen by artificial infection (Yan et al., 2001). The sample was stored at -80°C in physiological saline with 10% glycerol. The bacteria were cultured on tryptic soy broth agar (TSA) supplemented with 2% NaCl at 28°C and challenged with chemical stresses including Cu (50 mg/L), Pb (100 mg/L), Hg (50 mg/L), and low pH (pH = 5). The control group was cultured on a normal TSA slant (pH = 7). Three replicates were included for each treatment.

### High-Throughput Deep Sequencing

Head-on comparison of RNA-seq with microarrays has shown that RNA-seq has negligible technical variability, making it possible to obtain a reliable estimate of gene expression without replicate analysis (Reddy et al., 2012). Therefore, we applied RNA-seq and performed the analysis without replicates.

Extracted RNA samples collected from pooled bacteria (*n* = 3) were used for the generation of cDNA libraries. Total RNA was extracted from the bacteria using TRIzol (Invitrogen, Carlsbad, CA, USA) according to the manufacturer’s protocol. The RNA samples were subjected to rRNA depletion using the Ribo-Zero (gram-negative bacteria) kit (Epicentre, Madison, WI, USA) according to the manufacturer’s instructions. All mRNAs were broken into short (200-nt) fragments using fragmentation buffer. First-strand cDNA was generated using random hexamer-primed reverse transcription, followed by the synthesis of second-strand cDNA using RNase H and DNA polymerase I. The cDNA fragments were purified using the QIA Quick PCR extraction kit (Qiagen, Valencia, CA, USA). These purified fragments were washed with EB buffer for end reparation poly(A) addition and ligated to sequencing adapters. After agarose gel electrophoresis and extraction of the cDNA from the gels, the cDNA fragments (200 bp ± 25 bp) were purified using a MiniElute PCR Purification Kit (Qiagen, Valencia, CA, USA) and enriched by PCR to construct the final cDNA library. The cDNA library was sequenced using the Illumina sequencing platform (Illumina HiSeq^TM^ 2000, Illumina, San Diego, CA, USA). The processing of original images of sequences, base-calling, and quality value calculations were performed using the Illumina GA Pipeline (version 1.6), from which 100-bp paired-end reads were obtained. A Perl program was written to select clean reads by removing low-quality sequences (more than 50% of the bases with a quality lower than 20 in one sequence), reads with more than 5% N bases (unknown bases) and reads containing adaptor sequences. The clean reads were mapped to the reference genome (GCA_000354175.2) and gene sequences using SOAP2 (Li et al., 2009). Mismatches (≤5 bases) were allowed in the alignment.

The data were deposited in the NCBI Sequence Read Archive (SRA) and can be accessed through accession number SRP049226.

### Detection of Potential ncRNAs in *V. alginolyticus* Using Bioinformatics Analysis

Candidate ncRNAs were detected by high-throughput sequencing, with transcriptionally active regions (TARs) of lengths longer than 100 bp and average coverage depths larger than 2. TARs found in intergenic regions (100 bp away from the 3′-terminus of an upstream gene and 5′-terminus of a downstream gene) were blast searched against the nr database. If no match was found, the TARs were considered to be candidate ncRNAs. Two methods were applied for annotation of candidate ncRNAs: (1) sequence similarity and (2) consensus secondary structure. Candidate ncRNA sequences were searched against the sRNAMap, sRNATarBase and SIPHI databases based on sequence similarity (*e*-value < 0.00001). Annotated information of the candidate ncRNAs was obtained from the annotated information of the most similar sRNA in the database. Rfam, which is a collection of multiple sequence alignments and covariance models (CMs) covering many common ncRNA families, was introduced (Nawrocki et al., 2015). The main use of Rfam is as a source of RNA multiple alignments with consensus secondary structure annotation in a consistent format. In conjunction with the Infernal software package, Rfam CMs were used to search genomes or other DNA sequence databases for homology to known structural RNA families. ncRNA secondary structures were predicted using the Vienna RNA Package.

### ncRNA Expression Analysis

ncRNA expression was calculated using the RPKM (reads per kb per million reads) method (Mortazavi et al., 2008), which can eliminate the influence of different ncRNA lengths and sequencing discrepancies in the calculation of ncRNA expression and enable comparisons of differences in ncRNA expression among samples. We selected a threshold of false discovery rate (FDR) ≤0.001 and a minimum absolute fold difference of 2.0 between the control and stressed data sets. Then, we screened those ncRNAs showing a consistent changing trend (either a decrease or increase) among the stressed samples. The commonly changed ncRNAs were used for experiments.

### Quantitative RT-PCR Assay

ncRNAs and genes expression levels were verified by QPCR using Power SYBR Green PCR Master Mix (Applied Biosystems) in accordance with the manufacturer’s instructions. The results were normalized to 16S RNA (which showed invariant expression under the experimental conditions) and calculated using the 2^-ΔΔCt^ method (*n* = 3). The primers used for reverse transcription and QPCR are listed in **Supplementary Table S1**.

### ncRNA Target Prediction and Functional Analysis

IntaRNA was used to predict interactions between significantly changed ncRNAs and mRNAs. The scoring is based on the hybridization free energy and accessibility of the interaction sites in both molecules. The interaction has to contain an interaction seed, [i.e., a region of (nearly) perfect sequence complementarity] to facilitate the initiation of interaction. The features of this seed region are user-definable. Accessibility is defined as the free energy required to unfold the interaction site in each molecule. For calculation of these unfolding energies, global folding of the ncRNA and local folding of the mRNA are assumed. Finally, the target genes were used for gene ontology (GO) and KEGG pathway analyses.

### Stable Gene Silencing

To investigate the function of the ncRNAs, stable gene silencing was performed. The pACYC184 vector containing a short hairpin (sh) RNA (comprising the entire ncRNA sequence is listed in **Supplementary Table S2**) and a non-target shRNA as a control (5′- TTC TCC GAA CGT GTC ACG TTT -3′) were used for stable gene silencing. The vector was transferred into SM10 by electrotransformation and then transferred by conjugation from SM10 to *V. alginolyticus*. Chloromycetin was used to screen the stable silenced clones, which were used for RNA extracts and *in vitro* adhesion assay.

### Mucus Preparation

Healthy *P. crocea* were obtained from marine cage cultures, Ningde, Fujian Province, China. Skin mucus was prepared using a method modified from a previous study (Kong et al., 2015). The fish were washed with sterile phosphate buffered saline (PBS; 0.01 mol/L, pH 7.2). The skin mucus was harvested by scrapping the surface of the skin with a plastic spatula to remove the mucus gel layer covering the skin; the mucus gel was then homogenized in PBS. The mucus preparations were centrifuged twice at 20,000 *g* and 4.0°C for 30 min to remove particulate materials. The final supernatant was filtered through 0.45- and 0.22- μm pore filters. The mucus samples were adjusted to 1.0 mg protein/mL PBS. The protein concentration was determined using the method of Bradford (1976).

### *In Vitro* Adhesion Assay

The bacterial adhesion assay was conducted following the method described by Kong et al. (2015). Briefly, 50 μL mucus suspensions were spread evenly onto 22 mm^2^ glass slides and fixed with methanol for 20 min after the mucus was dry. Then, 1.0 mL aliquots of bacterial suspensions (10^8^ CFU/mL) was placed on the mucus-coated glass slides, incubated moistly at 25°C for 2.0 h, and washed thoroughly five times with PBS. Finally, the slides were fixed with 4.0% methanol for 30 min, dyed with crystal violet for 3.0 min, and bacteria were counted under a microscope (1000×). Three trials were conducted for each group and 20 microscope visions were selected.

### Data Processing

The results are reported as the means ± SE. The data were analyzed with one-way ANOVA followed by Dunnett’s multiple comparison test using the SPSS 13.0 software. A value of *P* < 0.05 indicated a significant difference.

## Results

### Basic Data Obtained

The candidate ncRNAs obtained by high-throughput sequencing and identified using the nr database and their basic data are listed in **Supplementary Tables S3**–**S5**. The lengths of most of these ncRNAs are in the range of 100–600 nt, with the predominant lengths in the range of 100–120 nt (**Supplementary Figure S1A**). Five samples exhibited similar ncRNA length distributions (**Supplementary Figure S1B**), which indicated the reliability of the sequencing.

### Differential Expression of ncRNAs

**FIGURE 1:**
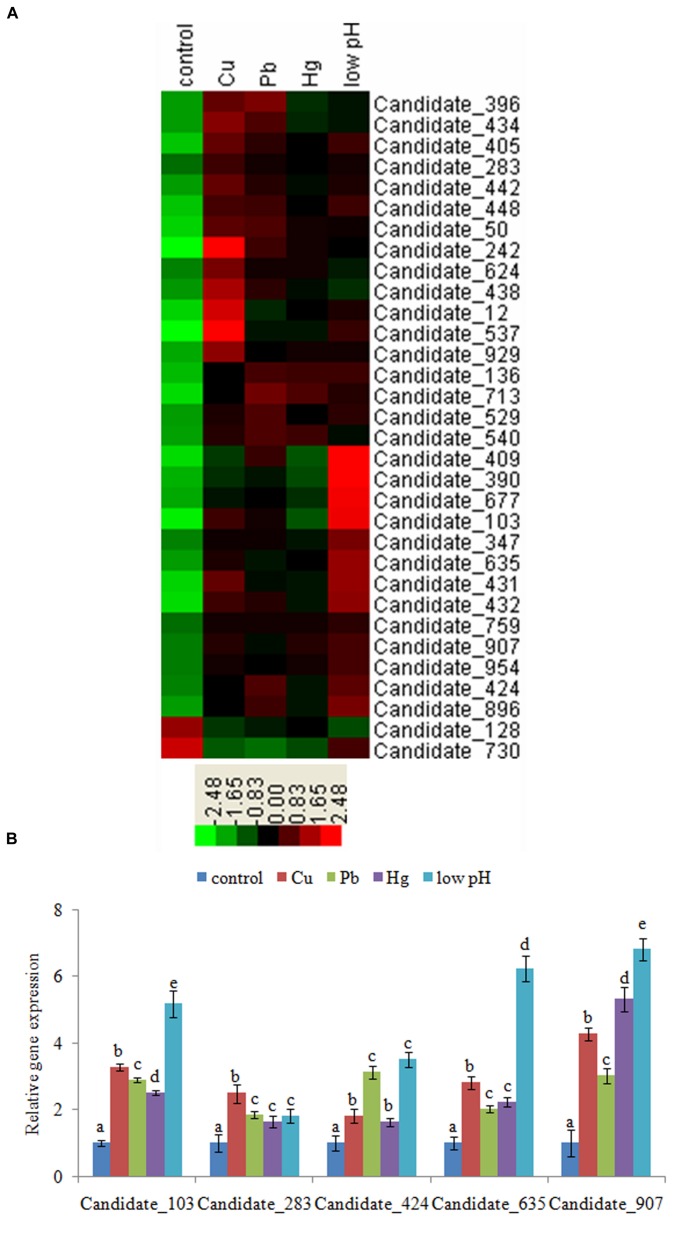
**(A)** Hierarchical clustering of commonly changed ncRNAs. Green and red indicate decreased and increased expression, respectively. Transcripts were clustered by hierarchical clustering using the complete linkage algorithm and Pearson correlation metric in R. **(B)** Validation of the results of high-throughput sequencing. QPCR analysis of the expression of 5 novel ncRNAs chosen from the 32 commonly changed ncRNAs was performed. The means of treatments not sharing a common letter are significantly different at *P* < 0.05 (Dunnett’s).

We selected a threshold of FDR ≤ 0.001 and a minimum absolute fold difference of 2.0 between the control and stressed data sets (Audic and Claverie, 1997). After Cu treatment, 255 ncRNAs were significantly altered (**Supplementary Table S5**), with 29 being down-regulated and 226 up-regulated. After Pb treatment, 177 ncRNAs were significantly changed (**Supplementary Table S5**); 48 were down-regulated and 129 up-regulated. A total of 157 ncRNAs were significantly changed after Hg treatment (**Supplementary Table S5**): 60 were down-regulated, and 97 were up-regulated. After low-pH treatment, 494 ncRNAs were significantly changed (**Supplementary Table S5**), with 34 and 460 being down- and up-regulated, respectively. We then screened those ncRNAs showing a consistent changing trend (either a decrease or increase) among the stressed samples. These approaches finally yielded 2 down-regulated and 30 up-regulated ncRNAs from the sequencing results. These 32 commonly changed ncRNAs were hierarchically clustered and used to produce a heat map (**Figure 1A**) according to the method of Eisen et al. (1998). These ncRNAs were used for further research.

### Validation of the Results of High-Throughput Sequencing

To validate the sequencing results, we performed QPCR on Candidate_103, Candidate_283, Candidate_424, Candidate_635, and Candidate_907. The QPCR results matched with the sequencing results: Cu, Pb, Hg, and low-pH treatment significantly up-regulated the expression of Candidate_103 (by 3.29-, 2.89-, 2.51-, and 5.19-fold, respectively), Candidate_283 (by 2.49-, 1.85-, 1.65-, and 1.82-fold, respectively), Candidate_424 (by 1.81-, 3.13-, 1.62-, and 3.51-fold, respectively), Candidate_635 (by 2.82-, 2.02-, 2.23-, and 6.24-fold, respectively), and Candidate_907 (by 4.28-, 3.03-, 5.32-, and 6.81-fold, respectively; **Figure 1B**). These results reinforced the reliability of the sequencing data.

### Functional Analysis of ncRNAs

We aligned the 32 commonly changed ncRNAs with known sRNA databases based on sequence similarity and consensus secondary structures. We identified 24 ncRNAs with clear characteristics (**Supplementary Table S6**), which might provide insight into the functions of these ncRNAs.

To examine the potential targets of the 32 commonly changed ncRNAs, their mRNA targets were computationally identified using the IntaRNA software (**Supplementary Table S7**). The target genes of the up-regulated ncRNAs included the type IV pilus assembly protein PilQ (K02666), type IV pilus assembly protein PilM (K02662), flagellar motor switch protein FliM (K02416), and chemotaxis protein MotB (K02557), which are associated with bacterial adhesion. In our previous study (Kong et al., 2015), we presented a transcriptome analysis of *V. alginolyticus* cultured under the same conditions used in the present study and found that Cu, Pb, Hg, and low-pH treatment significantly down-regulated the expression of *PilQ* (by 4.03-, 3.19-, 2.32-, and 2.16-fold, respectively), *PilM* (by 3.73-, 3.64-, 2.16-, and 3.71-fold, respectively), *FliM* (by 3.51-, 2.69-, 4.03-, and 2.49-fold, respectively), and *MotB* (by 3.04-, 2.12-, 2.05-, and 2.29-fold, respectively) (**Supplementary Table S7**).

**FIGURE 2:**
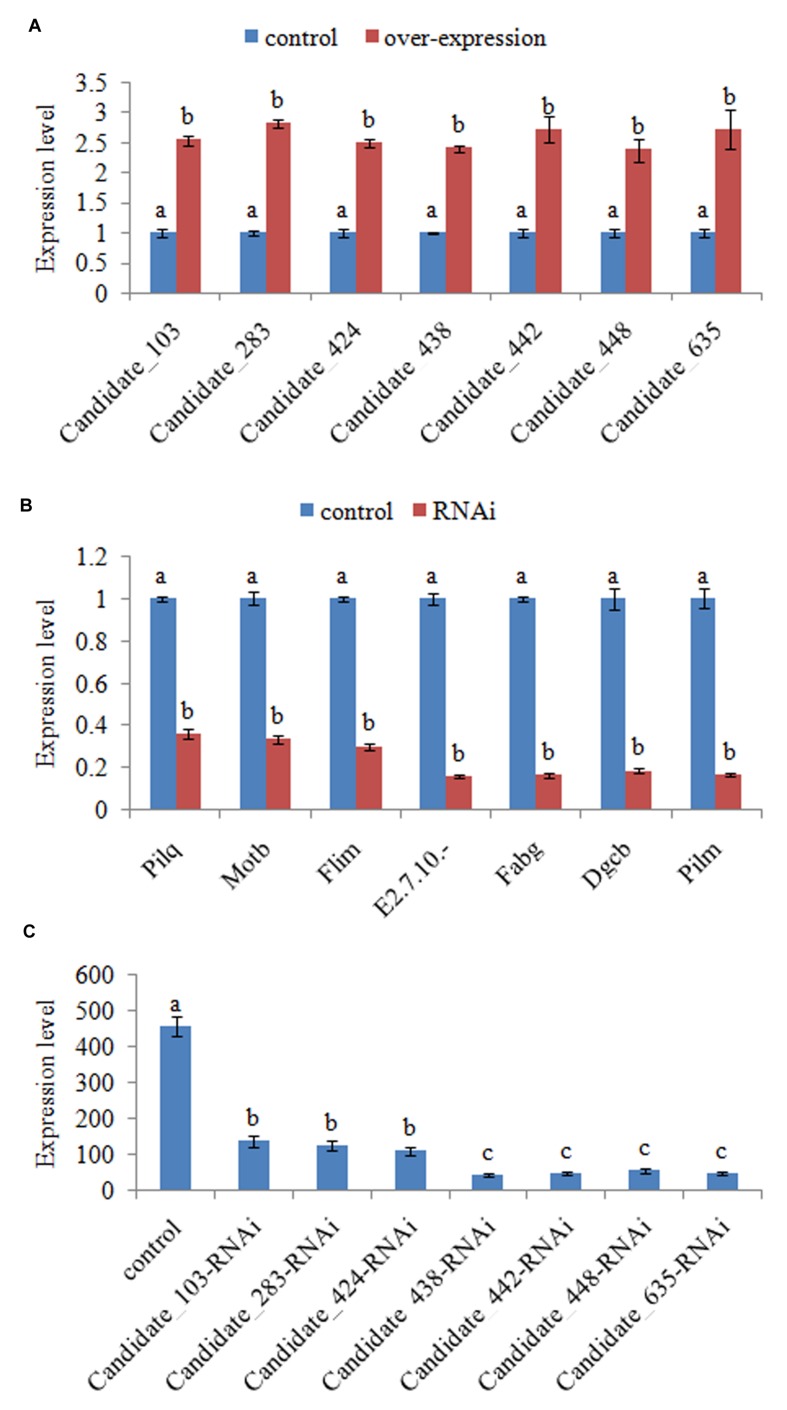
**Functional characterization of ncRNAs during adhesion. (A)** QPCR analysis of the expression of non-coding (nc) RNAs (Candidate_103, Candidate_283, Candidate_424, Candidate_438, Candidate_442, Candidate_448, and Candidate_635) in stable silenced clones. Data are presented as the mean ± SD (*n* = 6). Means of treatments not sharing a common letter are significantly different at *P* < 0.05. **(B)** QPCR analysis of the expression of target genes after stable gene silencing. Data are presented as the mean ± SD (*n* = 6). Means of treatments not sharing a common letter are significantly different at *P* < 0.05. **(C)** The capacity of stable silenced *V. alginolyticus* to adhere to mucus. Data are presented as the mean ± SD (*n* = 3). Means of treatments not sharing a common letter are significantly different at *P* < 0.05, as assessed using one-way ANOVA followed by the Dunnett’s test.

Interestingly, there seems to be a co-expression pattern between some of the commonly changed ncRNA (including Candidate_103, Candidate_283, Candidate_424, Candidate_438, Candidate_442, Candidate_448, Candidate_635, and Candidate_907) and their target genes. What’s more, the co-expression seems to be negative. Therefore, these ncRNAs might reduce adhesion by reducing the expression of their target genes. In order to validate this, stable gene silencing and the following *in vitro* adhesion assay were performed. Our results of QPCR showed that the expression levels of ncRNAs in stable silenced clones are significantly increased by 2.54-, 2.81-, 2.49-, 2.41-, 2.71-, 2.38-, and 2.72-fold, respectively (**Figure 2A**). This reinforces the reliability of stable gene silencing. The expression level of the target genes was also detected. The results of QPCR showed that the target genes are significantly reduced in stable silenced clones by 2.76-, 2.97-, 3.32-, 6.23-, 6.03-, 5.34-, and 5.90-fold, respectively (**Figure 2B**). The reduction of target genes indicates that the entire ncRNA sequence could decrease the expression of their target genes. The adhesion ability of stable silenced clones was also detected. Our results showed that approximately 458 cells/vision control *V. alginolyticus* adhered to the slides, while the numbers of adherent bacteria of Candidate_103-, Candidate_283-, Candidate_424-, Candidate_438-, Candidate_442-, Candidate_448-, and Candidate_635-RNAi *V. alginolyticus* were 136, 124, 108, 43, 46, 56, and 48 cells/vision, respectively (**Figure 2C**). This means the adhesion ability of *V. alginolyticus* was reduced by 3.37-, 3.69-, 4.24-, 10.65-, 9.96-, 8.18-, and 9.54-fold in stable silenced clones, which also demonstrates that the adhesion ability of stable silenced clones is significantly impaired. This further proves that these ncRNAs can negatively control the adhesion process of *V. alginolyticus* by negatively controlling the transcription of their target genes.

Gene ontology was introduced into the analysis to reveal functions significantly associated with the predicted target gene candidates of the altered ncRNAs. Some of the corresponding biological processes were interesting, such as “cell motility,” “response to chemical stimulus,” and “establishment of localization.” These processes might be associated with the decline in bacterial adhesion caused by stress (**Supplementary Table S8**).

To elucidate the biological functions of the changed ncRNAs, KEGG analysis was performed for the predicted ncRNA target genes. When gene ontology categories were assigned to these targets (**Supplementary Figures S2**–**S5**), nine molecular function categories predominated; the exception was the Pb-treated group, in which only seven molecular function categories predominated. Two of the most highly represented molecular function categories were “binding” and “catalytic activity.” Fifteen biological processes were identified, except in the Pb-treated group, for which 16 biological processes were identified. The two most frequent biological processes were “cellular process” and “metabolic process,” followed by “localization process,” “single-organism process,” and “establishment of localization.” Finally, there were ten major cellular component classes (only eight were found in the Pb- and low-pH-treated groups), with the two most abundant cellular components being “cell” and “cell part.” This analysis suggested that the ncRNA targets are mainly involved in biological regulation, cellular process, localization, establishment of localization and metabolic processes.

## Discussion

Although the length distributions of the *V. alginolyticus* ncRNAs in the five samples were similar, the expression levels of the ncRNAs were quite different. The results indicated that environmental conditions do affect the expression of ncRNAs. Majdalani et al. (2005) also reported the possibility of an unidentified environmental condition that enhanced the expression of a regulatory RNA. ncRNAs can modulate gene expression, including positive and negative regulation of mRNA stability and negative regulation of mRNA translation, in the post-transcription regulation of target mRNAs during infection and can also regulate phenotypes through certain biological processes (Liu et al., 2012). For example, Massé et al. (2007) found that ncRNAs such as RyhB control iron metabolism in *E. coli*, *V. cholerae*, and *Shigella flexneri*, and Winkler (2005) reported a role for ncRNAs in bacterial metabolic control. GcvTis is reported to be a glycine-sensing riboswitch that regulates glycine export and catabolism genes (Mandal et al., 2004). However, at present, little is known about the role of ncRNAs in the bacterial adhesion process. According to our previous study (Kong et al., 2015), *V. alginolyticus* exhibits a significant decrease in adhesion to fish mucus by 1.60-, 1.65-, 1.69-, and 2.31-fold when cultured under Cu, Pb, Hg, and low pH. In the present study, culturing *V. alginolyticus* under different stress conditions resulted in significant differences in ncRNA expression. These results indicate that ncRNA expression might be associated with the adhesion capacity of the bacteria.

Among the four stress groups in our previous study (Kong et al., 2015), the low-pH-treated group exhibited the maximal decline in adhesion, indicating that acidic conditions might have a stronger inhibiting effect on *V. alginolyticus* adhesion than heavy metal stresses. Interestingly, the number of significantly altered ncRNAs was higher in the low-pH-treated group compared to the other stress groups. This coincidence also indicates that perturbation of ncRNA expression might be one of the explanations for the decline in adhesion.

In the present study, 32 ncRNAs among four stressed samples showed a common change, with all four stressed samples exhibiting a decrease in bacterial adhesion. Therefore, these ncRNAs with a common change might be related to bacterial adhesion. When the comparison was performed between only one stressed sample and one control sample, 1083 ncRNAs showed differential expression. By considering the four stressed samples together, we identified only 32 ncRNAs, which greatly narrowed the screening scope of candidate functional ncRNAs.

The target genes of the up-regulated ncRNAs Candidate_103 and Candidate_635 are C408_2997 and C408_3001, which are the KEGG K02666//type IV pilus assembly protein PilQ and K02662//type IV pilus assembly protein PilM, respectively. According to our previous study (Kong et al., 2015), the expression of PilQ and PilM are significantly down-regulated by Cu, Pb, Hg, and low-pH treatment.

In several bacterial species (i.e., *E. coli*, *Pseudomonas aeruginosa*, *V. cholerae*, and *Salmonella enteritidis*), adhesins are present at the tips of complex cell-surface structures called pili or fimbriae, which extend from the outer cell membrane (Soto and Hultgren, 1999). Pili are classified as P, type I, type IV, and curli, and each has a distinct structural organization and assembly mechanism. Type IV pili are polymeric fibers that protrude from the cell surface and play a critical role in adhesion and invasion by pathogenic bacteria. *P. aeruginosa*, which is gram-negative, opportunistic pathogen, utilizes polar type IV pili (T4P) for twitching motility and adhesion in the environment and during infection (Jain et al., 2012). The secretion of type IV pilus components across the periplasm and outer membrane is mediated by the specialized secretin protein PilQ (Berry et al., 2012), a protein that is required for the assembly of type IV fimbriae in *P. aeruginosa.* A number of adhesins are involved in *P. aeruginosa* attachment. The PilM protein is required for competency and pilus biogenesis.

The target genes of the up-regulated ncRNAs Candidate_283 and Candidate_424 are the chemotaxis protein (MotB) and flagellar motor switch protein (FliM), respectively. MotB and FliM are closely associated with bacterial motility. The process of adhesion of bacteria is connected to the movement of bacteria in response to a chemical stimulus. According to our previous study (Kong et al., 2015), *MotB* and *FliM* expression is significantly down-regulated by Cu, Pb, Hg, and low-pH treatment. These results indicated that stresses might affect adhesion by perturbing bacterial motility, while Candidate_283 and Candidate_424 might be key regulators.

In addition to pili, which microorganisms use to firmly adhere to specific host cells, the process of bacterial adhesion is associated with characteristics of the bacterial cells themselves. These characteristics include velocity of movement, tropism, and hydrophobicity (Cheng et al., 2009). Motility in most bacterial species depends on a sophisticated molecular machine called the flagellum. Protein MotB is required for rotation of the flagellar motor, and it may be a linker that fastens the torque-generating machinery to the cell wall. The motor protein MotB converts an ion gradient (a proton gradient for most bacteria) into rotational energy of basal body components. These components are connected to the rod structure and then to the filament of the flagellum via a flexible hook. The flexible hook operates like a propeller (Rajagopala et al., 2007).

FliM is required for the assembly and function of flagella, which is important for bacterial motility (Toker et al., 1996). Interestingly, some researchers believe that some bacteria can sense the existence of a surface by using a mechanism call flagellar “surface mechanosensing,” which plays an important role in bacterial adhesion (Dang and Lovell, 2016).

The target gene of the up-regulated ncRNA Candidate_907 is the methyl-accepting chemotaxis protein (MCP), which encoding transmembrane receptor varies in the molecules that it detects. MCP is closely associated with bacterial chemotaxis, which plays an important role in adhesion. According to our previous study (Kong et al., 2015), MCP expression was significantly down-regulated by Cu, Pb, Hg, and low-pH treatment. Therefore, stresses might affect adhesion by perturbing bacterial chemotaxis, while Candidate_907 might be key regulator.

Many motile bacteria sense and respond to changes in their environment using a variation of the *E. coli* chemotaxis paradigm. The two-component signal transduction pathway responds to changes in the concentration of specific ligands via transmembrane MCPs and then causes a change in the direction of swimming that is associated with bacterial adhesion.

RNA-mediated regulation is quite different from protein-mediated regulation, which may have a number of potential advantages for bacteria (Beisel and Storz, 2010). First, ncRNAs do not require translation and occupy a much lower proportion of the genome. Second, ncRNAs can have multiple targets and multiple ncRNAs can regulate a single target under different conditions (Repoila et al., 2003; Waters and Storz, 2009). Finally, ncRNAs have dramatically different half-lives, ranging from 2.0 to >30 min (Vogel et al., 2003). These differences could potentially affect the duration of regulation mediated by ncRNAs.

These ncRNAs and target genes associated with bacterial adhesion must play key roles in the decline of adhesion ability caused by chemical stress. Because these ncRNAs repress rather than promote virulence in *V. alginolyticus*, adhesion repression by some methods affecting the expression of ncRNAs could be considered the basis of a strategy for therapeutic intervention of *V. alginolyticus* pathogenicity. However, further research is necessary.

## Conclusion

The present study demonstrated that treatment of *V. alginolyticus* with chemical stress could induce changes in the ncRNA expression profile. To the best of our knowledge, this is the first analysis of differentially expressed ncRNAs in *V. alginolyticus* following chemical stress. Our results demonstrated that some of the altered ncRNAs are involved in bacterial adhesion, which validates our previous results and offers new insight into the mechanism(s) underlying the regulation of *V. alginolyticus* adhesion. However, further research is still necessary.

## Author Contributions

LH participated in the microbiology studies, carried out the analysis and interpretation of data immunoassays, participated in the sequence alignment and drafted the manuscript. JH carried out the microbiology studies, participated in the sequence alignment and drafted the manuscript. YS conceived of the study, participated in the sequence alignment. YQ participated in the design of the study and performed the statistical analysis. WK participated in the microbiology studies. LZ performed the statistical analysis. YM participated in the design of the study and performed the statistical analysis. XX participated in the design of the study and performed the statistical analysis. ML participated in the microbiology studies. JZ participated in the microbiology studies. QY conceived of the study, participated in its design and coordination, and participated in the sequence alignment and drafted the manuscript. All authors read and approved the final manuscript.

## Conflict of Interest Statement

The authors declare that the research was conducted in the absence of any commercial or financial relationships that could be construed as a potential conflict of interest. The handling Editor declared a shared affiliation, though no other collaboration, with several of the authors and states that the process nevertheless met the standards of a fair and objective review.

## Supplementary material

FIGURE S1 **Length distribution of candidate ncRNAs. (A)** Length distribution of all ncRNAs as a whole. **(B)** Length distribution of the ncRNAs of each sample.

FIGURE S2 **Gene ontology analysis of target genes of differentially expressed ncRNAs in the Cu-stressed group**.

FIGURE S3 **Gene ontology analysis of target genes of differentially expressed ncRNAs in the Pb-stressed group**.

FIGURE S4 **Gene ontology analysis of target genes of differentially expressed ncRNAs in the Hg-stressed group**.

FIGURE S5 **Gene ontology analysis of target genes of differentially expressed ncRNAs in the low-pH-stressed group**.

TABLE S1 **Primer sequences used in QPCR analysis**.

TABLE S2 **ncRNA sequences**.

TABLE S3 **Location of candidate ncRNAs**.

TABLE S4 **Reads per kb per million reads (RPKM) of candidate ncRNAs**.

TABLE S5 **Differential expression of ncRNAs under stress treatments**.

TABLE S6 **Alignment based on similarity of sequences and consensus secondary structure of commonly changed ncRNAs**.

TABLE S7 **Identification of target genes of commonly changed ncRNAs**.

TABLE S8 **Gene ontology analysis of target genes of commonly changed ncRNAs**.
