# Supplementary figures and images for: Genome-Wide Detection of Predicted Non-coding RNAs Related to the Adhesion Process in Vibrio alginolyticus Using High-Throughput Sequencing
Source: Front Microbiol. 2016 Apr 28;7:619. doi: 10.3389/fmicb.2016.00619 (PMC4848308; doi:10.3389/fmicb.2016.00619)

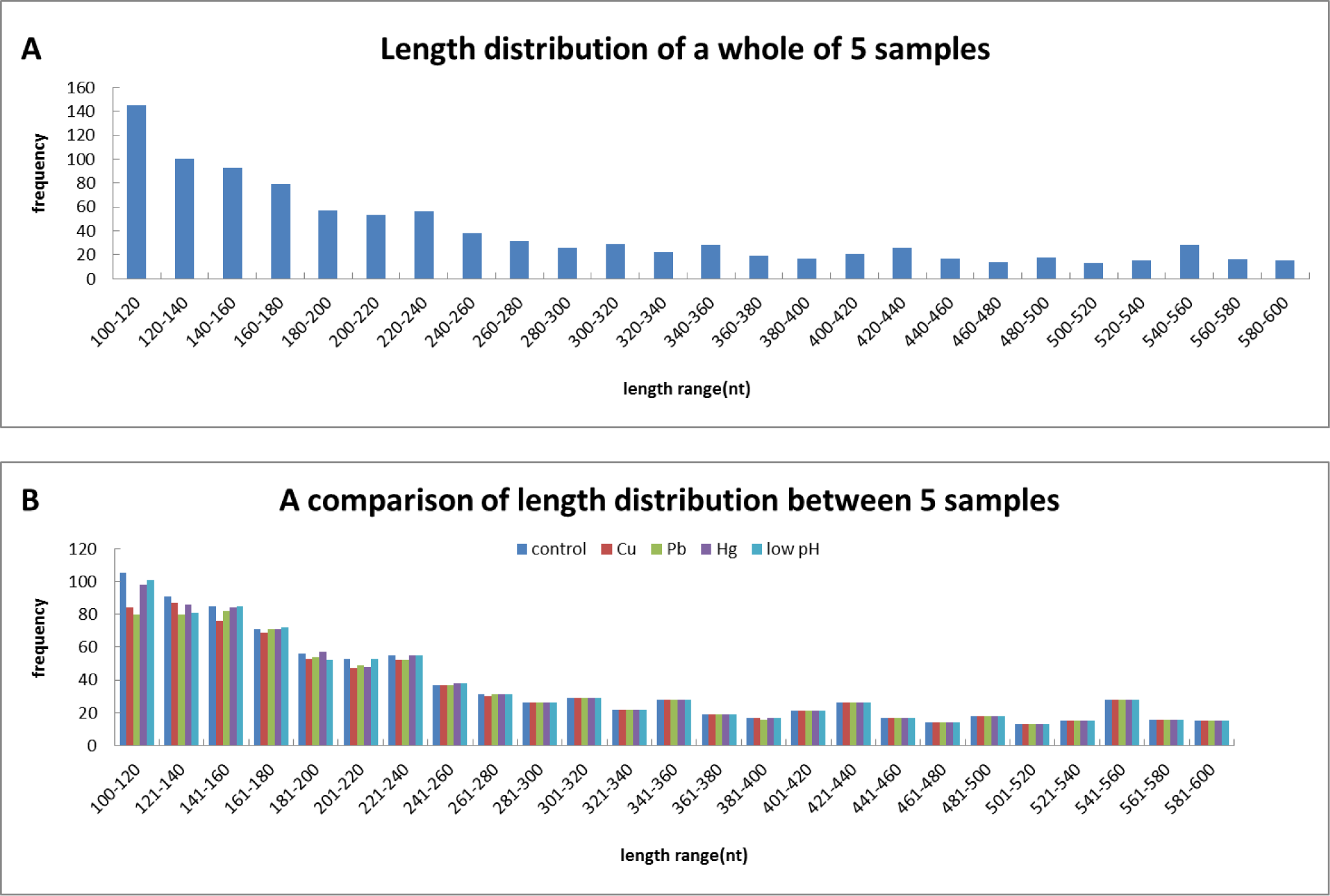

Supplement: FIGURE S1 — Length distribution of candidate ncRNAs. (A) Length distribution of all ncRNAs as a whole. (B) Length distribution of the ncRNAs of each sample. [file Image_1.TIF]

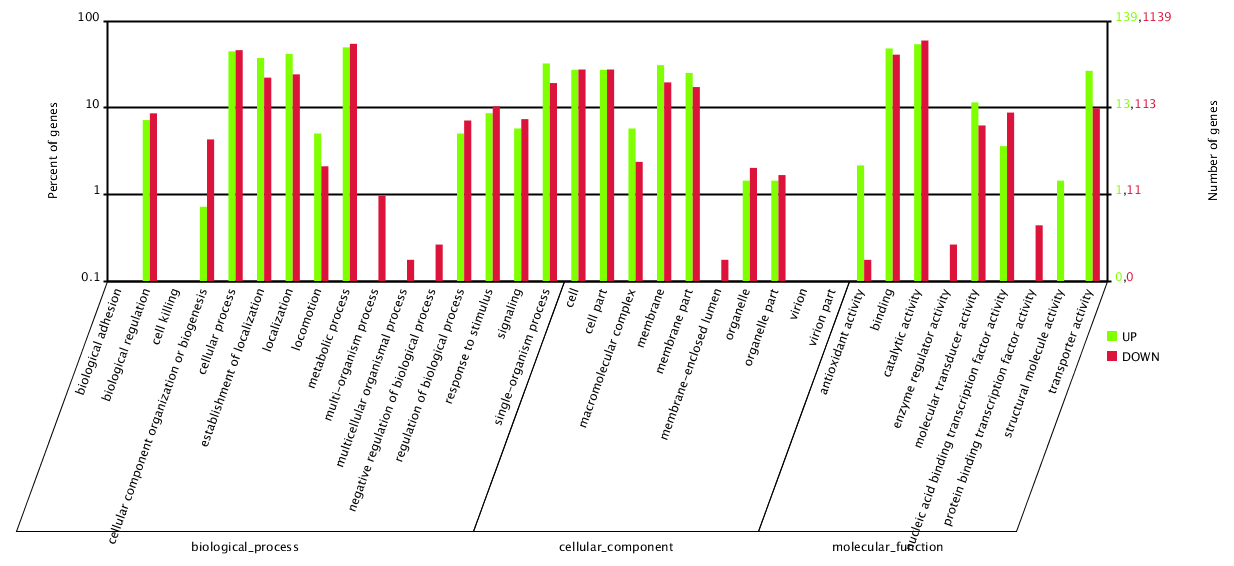

Supplement: FIGURE S2 — Gene ontology analysis of target genes of differentially expressed ncRNAs in the Cu-stressed group. [file Image_2.TIF]

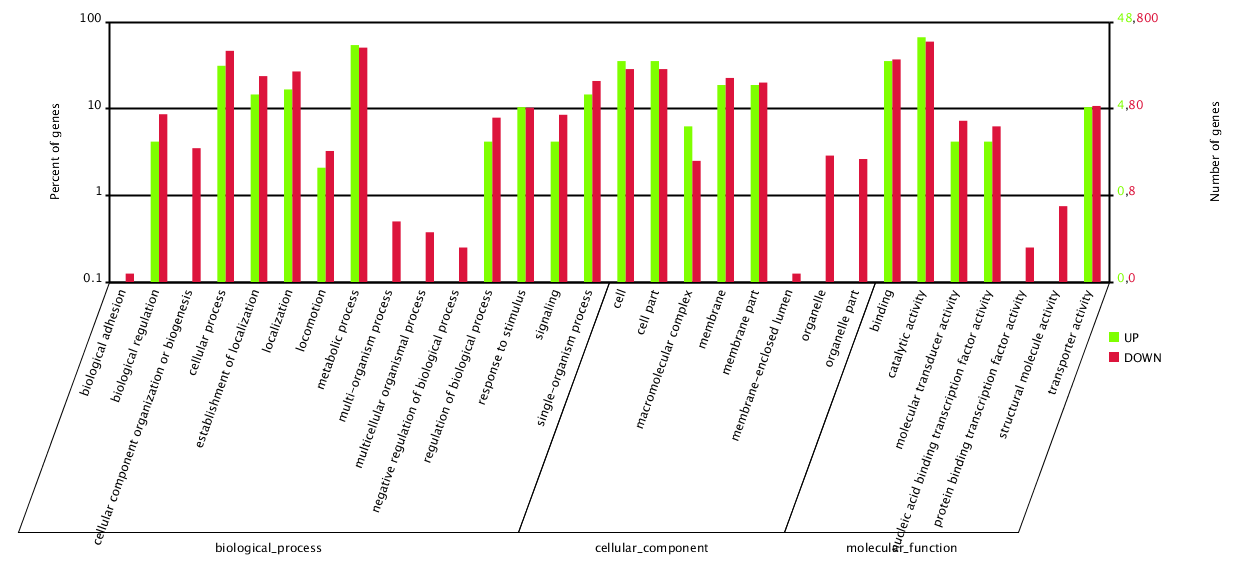

Supplement: FIGURE S3 — Gene ontology analysis of target genes of differentially expressed ncRNAs in the Pb-stressed group. [file Image_3.TIF]

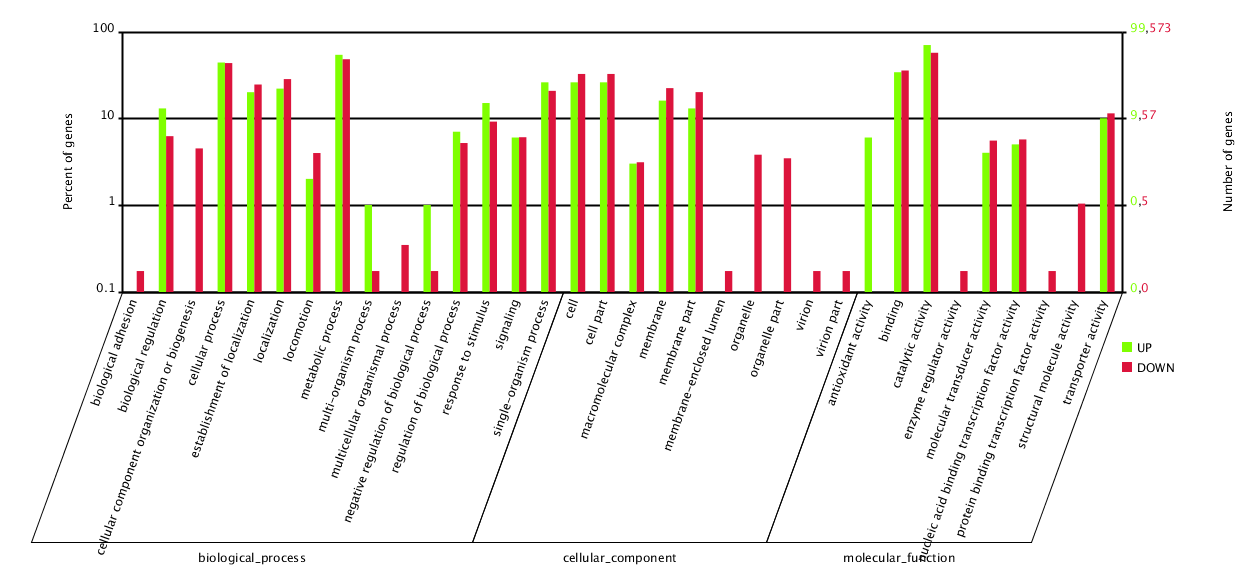

Supplement: FIGURE S4 — Gene ontology analysis of target genes of differentially expressed ncRNAs in the Hg-stressed group. [file Image_4.TIF]

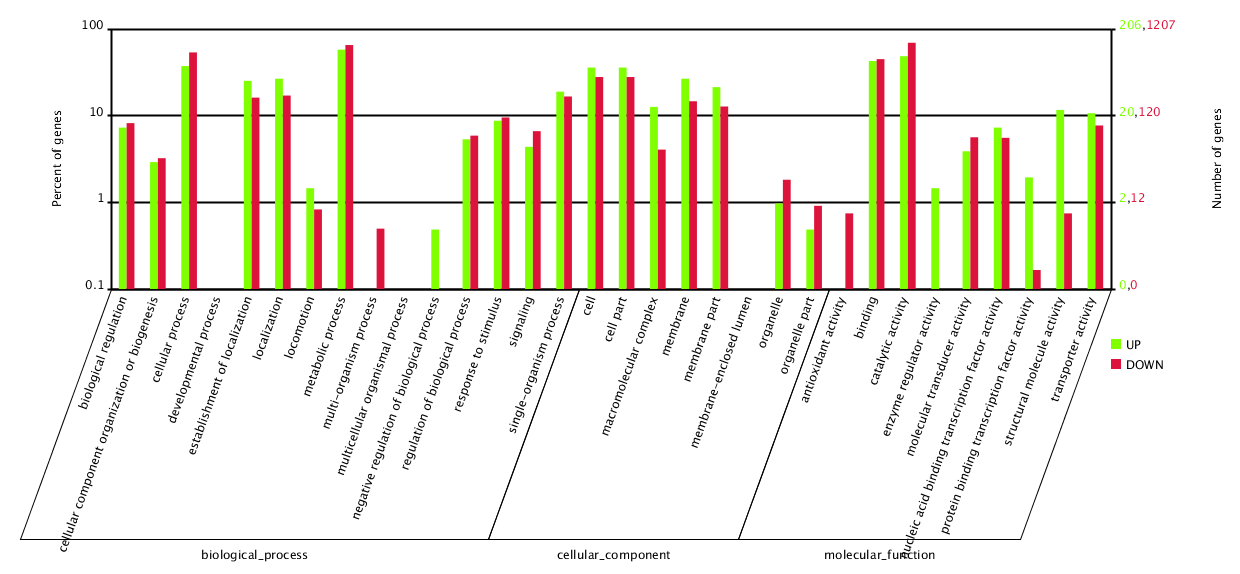

Supplement: FIGURE S5 — Gene ontology analysis of target genes of differentially expressed ncRNAs in the low-pH-stressed group. [file Image_5.TIF]
