# Supplementary material for: Genome-Wide Detection of Predicted Non-coding RNAs Related to the Adhesion Process in Vibrio alginolyticus Using High-Throughput Sequencing
Source: Front Microbiol. 2016 Apr 28;7:619. doi: 10.3389/fmicb.2016.00619 (PMC4848308; doi:10.3389/fmicb.2016.00619)
Supplement: TABLE S1 — Primer sequences used in QPCR analysis. [file Table_1.DOCX]

**Table 1. Primers for QPCR**

| **ncRNA** | **Primers for reverse transcription** |
| --- | --- |
| Candidate_103 | 5'-GTCGTATCCAGTGCAGGGTCCGAGGTATTCGCACTGGATACGACATAGAGG-3' |
| Candidate_283 | 5'-GTCGTATCCAGTGCAGGGTCCGAGGTATTCGCACTGGATACGACAGGAGGG-3' |
| Candidate_424 | 5'-GTCGTATCCAGTGCAGGGTCCGAGGTATTCGCACTGGATACGACCTTACCA-3' |
| Candidate_635 | 5'-GTCGTATCCAGTGCAGGGTCCGAGGTATTCGCACTGGATACGACTGCACGA-3' |
| Candidate_907 | 5'-GTCGTATCCAGTGCAGGGTCCGAGGTATTCGCACTGGATACGACCAGTGCA-3' |
| **ncRNA** | **Primers for QPCR** |
| 16S RNA | 5'-GGGGAGTACGGTCGCAAGAT-3' (F)  5'-CGCTGGCAAACAAGGATAAGG-3' (R) |
| Candidate_103 | 5'-TACCGTCTTTTACACAGTCTTT-3' (F)  5'-GCAGGGTCCGAGGTATTC-3' (R) |
| Candidate_283 | 5'-GCGTTATCCCTCCTGT-3' (F)  5'-GCAGGGTCCGAGGTATTC-3' (R) |
| Candidate_424 | 5'-TGGATCTCGTTGACTGAACATT-3' (F)  5'-GCAGGGTCCGAGGTATTC-3' (R) |
| Candidate_438 | 5'-CGGGACGAGCGATTAG-3' (F)  5'-CGAGATTGCTTGACCTA-3' (R) |
| Candidate_442 | 5'-GATTGATCCTTGAGATT-3' (F)  5'-CGTGGGTATTTGCTTTC-3' (R) |
| Candidate_448 | 5'-TCTACTCCCATGCTCA-3' (F)  5'-TGTTACTCGTTAATTCGT-3' (R) |
| Candidate_635 | 5'-GGCCTATATCGATGCCGGTAA -3' (F)  5'-GCAGGGTCCGAGGTATTC-3' (R) |
| Candidate_907 | 5'-GTACCGTCTCTCTGCTGCGA -3' (F)  5'-GCAGGGTCCGAGGTATTC-3' (R) |
