# Supplementary material for: Genome-Wide Detection of Predicted Non-coding RNAs Related to the Adhesion Process in Vibrio alginolyticus Using High-Throughput Sequencing
Source: Front Microbiol. 2016 Apr 28;7:619. doi: 10.3389/fmicb.2016.00619 (PMC4848308; doi:10.3389/fmicb.2016.00619)
Supplement: TABLE S2 — ncRNA sequences. [file Table_2.DOCX]

**Table 2. ncRNA sequences.**

| **ncRNA:** Candidate_103 |
| --- |
| **ncRNA sequence:**  AAGAAGAAUGAAAUGCUCAAAGGAUGAGUCCGUUUUAUCGCCUCCAAGGAACCGAGAAAUCAACGAAUGUUUAAUUGAUUUAAAAGGUGAUAGUUAUGAAAGGUUUACCAAGUACAAUGUUCUGGAAUAGCAAGUCUGUUUACACCGGCAACUUUGUAUACCCAACAAGCUUUGGCUACUAAGUGAAGUCAAAGCAUGUGACUCGAACAGUUUGUUCACCCCUAUAAAUUGGAAUUUUUUUUCAGUGAUAGUGCUGACAUGAUUCGCCGCCACCCAAAUUGGGUGGCGUUUUUUUAUCUCCACUUUCUCUUUCUGACACAUUUUCUGCCAUCAAGGCUGGAUUCUAUUCUCCGUUCUUCAGUUUUAUCGAC |
| **ncRNA:** Candidate_283 |
| **ncRNA sequence:**  GCGTTATCCCTCCTTCTTGATAAGCACCCTCTATCGTTACGACAAATTTATATAGACAAACGTAGAGGGTGATTTCGTTATAGTAATAAGCAGAAGTAACCAGATACAGCGTAGGAAAGCACTCGAAAATGAGGTTGTTTTTTTGATGTGCAATGTGCCTATGATTGGTGTAAATCGATACGTTACAGCATCGTGGAAGGTGTATTTTCATCGGACACTAAACTCAGTCTAATCACGTAGTTTATTCCCATGTACCATGAGGTCACTCGGGTATAGACGTCTATCTGCTTTTATGACTTTGGGCTTCGCGTAATTGCTTGAATCAGGAGTCAAA |
| **ncRNA:** Candidate_424 |
| **ncRNA sequence:**  AATGAAATGAACGTAGGGCAGATGCCAAAAAGCCGCATCAAAAGATGCGGCTTTTTAAAAATGGCTCCTCCTACTGGACTCGAACCAGTGACCTGCGGATTAACAGTCCGTCGCTCTACCAACTGAGCTAAGGAGGAATTATTCGTGTATCGTGTTTAGCAATGACACCAAATAATGGTGCCTCGAGGCGGAATCGAACCACCGACACGAGGATTTTCAATCCTCTGCTCTACCGACTGAGCTATCGAGGCAAAAGAATGGTGCCGACTACCGGAATCGAACTGGTGACCTACTGATTACAAGTCAGTTGCTCTACCTACTGAGCTAAGTCGGCACACTTTATTCTTTGTGCTTTTGTCCGTGTTATAACATTTCGTTAAGACACCAACAAATCAAATTGTGGTGCCCGGAGGCGGAATCGAACCACCGACACGAGGATTTTCAATCCTCTGCTCTACCGACTGAGCTATCCGGGCGACGGAGCGCTATTAAACGGATTTTCCCGCTTTGCGTC |
| **ncRNA:** Candidate_438 |
| **ncRNA sequence:**  TCACCTTTAACTCGGGACGAGCGATTAGCGCTTGTCACTGATTAACACTAAGTCCAAATAGAGGGGTAGCTGTTGTGAACATAAACGCAACTCTGCTAGGTCAAGCAATCTCGTTTGCACTATTTGTGTGGTTCTGC |
| **ncRNA:** Candidate_442 |
| **ncRNA sequence:**  CGATCAAGCATCAGGATCGATTGATCCTTGAGATTTTACTCACACTACGTATAATCGCCCGGCCGGAATTTCGTACTCATTGAGTCATTGACACATTATGTGAGTGTGATTACAATTCCGCCTCTTTGTTGAGAGGCGTCGGTTGTCCTTCTCTATATAAAGAGCTTCTTTATATAAGAAAGCAAATACCCACGCCGGGTTTAACAAGAACCTAAAACTACTGATCAGTAAAGGTAATTATC |
| **ncRNA:** Candidate_448 |
| **ncRNA sequence:**  AATTAATCAAGATTAGCTCTACTCCCATGCTCAATATCTGGTTATACTAGATTGTTAATTTATTCAACCGTAATTACTCTTTATCGCCGTTGTTAGAGTTGAAAAACTCGGCATAGATGACGAATTAACGAGTAACAAGAGAATCGCGCGAACCTGACTT |
| **ncRNA:** Candidate_635 |
| **ncRNA sequence:**  TTTCTTTGTAACCCAACAGAGCGTAATTGTTGCCTGTGGGTTTAAGTACTACGGCTTTGAGGCTGTTATGGCCTATATCGATGCCGGTAACGATTAGCTTATCCATCTCTTACTCTCGCTCCGAAATTTCTTCGTGCAAAGGTTGTTATTAGCACCCAAATAAGAGTTAATATCTCTATGTGCTAAATTTTTGACCTAAGAGTACAAGGGTTTGCCGTTACACAGCCTCAACCAATCAGGGATTCTCCGGTGAAGTTCATAAAGCGTTTATTATTTTTAACATTGATTTGCATAATTCTTGGAGTCACCACAATATTTGGCTTCTATCAGTAC |
