# Supplementary material for: Genome-Wide Detection of Predicted Non-coding RNAs Related to the Adhesion Process in Vibrio alginolyticus Using High-Throughput Sequencing
Source: Front Microbiol. 2016 Apr 28;7:619. doi: 10.3389/fmicb.2016.00619 (PMC4848308; doi:10.3389/fmicb.2016.00619)
Supplement: TABLE S6 — Alignment based on similarity of sequences and consensus secondary structure of commonly changed ncRNAs. [file Table_6.DOCX]

**Table 6. Alignment based on similarity of sequences and consensus secondary structure of commonly changed ncRNAs.**

| **ncRNA** | **ID （Based on similarity of sequences）** | **ID （Based on consensus secondary structure）** |
| --- | --- | --- |
| Candidate_103 | Candidate_27_NC_009457 | Phe_leader |
| Candidate_12 |  | Thr_leader |
| Candidate_283 |  | mir-282 |
| Candidate_396 |  | PK-CuYV_BPYV |
| Candidate_405 |  | SNORD20 |
| Candidate_409 |  | snR51 |
| Candidate_424 | sRNAPU0151 | tRNA |
| Candidate_431 |  | mir-324 |
| Candidate_434 | Candidate_40_NC_004603 | RsmY |
| Candidate_438 |  | SNORD89 |
| Candidate_442 |  | mir-280 |
| Candidate_50 |  | snoU6-77 |
| Candidate_529 |  | rne5 |
| Candidate_537 |  | GlmZ_SraJ |
| Candidate_540 |  | ceN67 |
| Candidate_624 |  | mir-360 |
| Candidate_635 |  | ceN89 |
| Candidate_677 |  | mir-499 |
| Candidate_759 |  | snoR113 |
| Candidate_896 |  | class_I_RNA |
| Candidate_907 |  | mir-142 |
| Candidate_929 |  | sbcD |
| Candidate_954 |  | mir-12 |
| Candidate_128 |  | sR24 |
