# Supplementary material for: Genome-Wide Detection of Predicted Non-coding RNAs Related to the Adhesion Process in Vibrio alginolyticus Using High-Throughput Sequencing
Source: Front Microbiol. 2016 Apr 28;7:619. doi: 10.3389/fmicb.2016.00619 (PMC4848308; doi:10.3389/fmicb.2016.00619)
Supplement: TABLE S7 — Identification of target genes of commonly changed ncRNAs. [file Table_7.DOCX]

**Table 7. Identification of target genes of commonly changed ncRNAs.**

| **ncRNA** | **Fold change** | | | | **Target** | **Fold change** | | | |
| --- | --- | --- | --- | --- | --- | --- | --- | --- | --- |
|  | **Cu** | **Pb** | **Hg** | **low pH** |  | **Cu** | **Pb** | **Hg** | **low pH** |
| Candidate_103 | 7.65 | 6.02 | 2.93 | 25.86 | Pilq | -4.03 | -3.19 | -2.32 | -2.16 |
| Candidate_136 | 3.47 | 5.58 | 5.40 | 5.54 | Hsdr | -1.18 | -1.34 | -1.33 | -2.39 |
| Candidate_242 | 93.04 | 18.72 | 13.94 | 11.79 | Etfdh | -1.12 | 1.49 | 2.68 | -1.18 |
| Candidate_283 | 3.23 | 2.40 | 2.15 | 2.36 | Motb | -3.04 | -2.12 | -2.05 | -2.29 |
| Candidate_390 | 2.42 | 2.87 | 2.05 | 30.28 | Pled | 1.12 | 1.02 | -2.68 | 1.31 |
| Candidate_405 | 7.71 | 5.17 | 3.72 | 5.85 | Ycjf | -4.38 | -2.18 | -1.81 | -1.22 |
| Candidate_409 | 3.06 | 6.54 | 2.53 | 38.75 | Lgt | -3.92 | -2.53 | -3.37 | 2.22 |
| Candidate_424 | 2.35 | 4.08 | 2.10 | 4.56 | Flim | -3.51 | -2.69 | -4.03 | -2.49 |
| Candidate_431 | 8.55 | 3.89 | 3.76 | 12.02 | Trpd | -1.31 | -1.05 | 1.15 | 1.25 |
| Candidate_432 | 6.71 | 5.56 | 3.72 | 11.42 | E2.3.1.- | -1.14 | 1.00 | 1.14 | 1.23 |
| Candidate_434 | 7.19 | 5.00 | 2.23 | 2.53 | Ompr | -2.04 | 1.35 | -1.51 | -9.05 |
| Candidate_438 | 8.88 | 3.78 | 2.55 | 2.06 | E2.7.10.- | -6.36 | -14.44 | -4.04 | -3.84 |
| Candidate_442 | 5.49 | 3.74 | 2.59 | 3.47 | Fabg | -3.36 | -4.31 | -2.26 | -3.76 |
| Candidate_448 | 6.01 | 5.76 | 3.95 | 5.70 | Dgcb | -3.16 | -3.96 | -2.09 | -3.48 |
| Candidate_50 | 7.74 | 6.97 | 4.88 | 4.65 | Aqpz | 1.87 | 1.43 | 3.67 | 1.22 |
| Candidate_529 | 3.35 | 4.89 | 2.87 | 3.86 | Ftra | -5.86 | -3.06 | 1.03 | -1.72 |
| Candidate_537 | 61.49 | 7.38 | 7.16 | 12.14 | Bett | -3.86 | -2.57 | -6.51 | -1.09 |
| Candidate_540 | 3.95 | 4.96 | 4.42 | 2.81 | Dgcb | -3.77 | -2.26 | -2.68 | -1.07 |
| Candidate_635 | 3.66 | 2.63 | 2.90 | 8.12 | Pilm | -3.73 | -3.64 | -2.16 | -3.71 |
| Candidate_677 | 2.72 | 3.14 | 2.32 | 16.75 | Parc | 1.10 | 1.12 | 1.10 | -1.11 |
| Candidate_759 | 2.51 | 2.45 | 2.52 | 2.92 | Ftsw | -1.50 | -1.16 | 1.08 | 1.77 |
| Candidate_896 | 2.82 | 4.37 | 2.60 | 6.61 | MANBA | -10.48 | -9.12 | -11.43 | 1.39 |
| Candidate_907 | 3.00 | 2.20 | 3.12 | 3.73 | MCP | -5.73 | -2.53 | -2.31 | -3.46 |
| Candidate_929 | 8.19 | 2.98 | 3.54 | 3.57 | Glgc | -3.06 | -2.85 | -1.41 | -3.58 |
| Candidate_128 | -3.95 | -3.25 | -2.62 | -4.54 | Tnaa | -7.33 | -4.08 | -2.38 | -13.83 |
