# Supplementary material for: Genome-Wide Detection of Predicted Non-coding RNAs Related to the Adhesion Process in Vibrio alginolyticus Using High-Throughput Sequencing
Source: Front Microbiol. 2016 Apr 28;7:619. doi: 10.3389/fmicb.2016.00619 (PMC4848308; doi:10.3389/fmicb.2016.00619)
Supplement: TABLE S8 — Gene ontology analysis of target genes of commonly changed ncRNAs. [file Table_8.DOCX]

**Table 8. GO analysis of target genes of commonly changed ncRNAs.**

| **Go term** | **Biological processes** |
| --- | --- |
| GO:0030031 | cell projection assembly |
| GO:0015031 | protein transport |
| GO:0006351 | transcription, DNA-dependent |
| GO:0007165 | signal transduction |
| GO:0009124 | nucleoside monophosphate biosynthetic process |
| GO:0006351 | transcription, DNA-dependent |
| GO:0023052 | signaling |
| GO:0048870 | cell motility |
| GO:0042221 | response to chemical stimulus |
| GO:0006351 | transcription, DNA-dependent |
| GO:0007165 | signal transduction |
| GO:0019222 | regulation of metabolic process |
| GO:0045226 | extracellular polysaccharide biosynthetic process |
| GO:0008610 | lipid biosynthetic process |
| GO:0006796 | phosphate-containing compound metabolic process |
| GO:0008152 | metabolic process |
| GO:0007165 | signal transduction |
| GO:0009124 | nucleoside monophosphate biosynthetic process |
| GO:0051234 | establishment of localization |
| GO:0023052 | signaling |
| GO:0006568 | tryptophan metabolic process |
